# Comparison of Landsat and Land-Based Phenology Camera Normalized Difference Vegetation Index (NDVI) for Dominant Plant Communities in the Great Basin

**DOI:** 10.3390/s19051139

**Published:** 2019-03-06

**Authors:** Keirith A. Snyder, Justin L. Huntington, Bryce L. Wehan, Charles G. Morton, Tamzen K. Stringham

**Affiliations:** 1 USDA-ARS, Great Basin Rangelands Research Unit, Reno, NV 89512, USA; b.wehan@gmail.com; 2 Desert Research Institute, Reno, NV 89512, USA; justin.huntington@dri.edu (J.L.H.); charles.morton@dri.edu (C.G.M.); 3 Department of Agriculture, Veterinary and Rangeland Science, University of Nevada, Reno, NV 89557 USA; tstringham@cabnr.unr.edu

**Keywords:** phenocams, Landsat, NDVI, semi-arid, cold desert, sagebrush, pinyon and juniper, meadows, StarDot cameras, camera-based repeat digital photography

## Abstract

Phenology of plants is important for ecological interactions. The timing and development of green leaves, plant maturity, and senescence affects biophysical interactions of plants with the environment. In this study we explored the agreement between land-based camera and satellite-based phenology metrics to quantify plant phenology and phenophases dates in five plant community types characteristic of the semi-arid cold desert region of the Great Basin. Three years of data were analyzed. We calculated the Normalized Difference Vegetation Index (NDVI) for both land-based cameras (i.e., phenocams) and Landsat imagery. NDVI from camera images was calculated by taking a standard RGB (red, green, and blue) image and then a near infrared (NIR) plus RGB image. Phenocam NDVI was calculated by extracting the red digital number (DN) and the NIR DN from images taken a few seconds apart. Landsat has a spatial resolution of 30 m^2^, while phenocam spatial resolution can be analyzed at the single pixel level at the scale of cm^2^ or area averaged regions can be analyzed with scales up to 1 km^2^. For this study, phenocam regions of interest were used that approximated the scale of at least one Landsat pixel. In the tall-statured pinyon and juniper woodland sites, there was a lack of agreement in NDVI between phenocam and Landsat NDVI, even after using National Agricultural Imagery Program (NAIP) imagery to account for fractional coverage of pinyon and juniper versus interspace in the phenocam data. Landsat NDVI appeared to be dominated by the signal from the interspace and was insensitive to subtle changes in the pinyon and juniper tree canopy. However, for short-statured sagebrush shrub and meadow communities, there was good agreement between the phenocam and Landsat NDVI as reflected in high Pearson’s correlation coefficients (*r* > 0.75). Due to greater temporal resolution of the phenocams with images taken daily, versus the 16-day return interval of Landsat, phenocam data provided more utility in determining important phenophase dates: start of season, peak of season, and end of season. More specific species-level information can be obtained with the high temporal resolution of phenocams, but only for a limited number of sites, while Landsat can provide the multi-decadal history and spatial coverage that is unmatched by other platforms. The agreement between Landsat and phenocam NDVI for short-statured plant communities of the Great Basin, shows promise for monitoring landscape and regional-level plant phenology across large areas and time periods, with phenocams providing a more comprehensive understanding of plant phenology at finer spatial scales, and Landsat extending the historical record of observations.

## 1. Introduction

Plant phenology is the timing of seasonal life cycles. Detecting plant phenology from land-based and satellite imagery has received increasing amounts of attention in the last two decades [1,2,3,4,5,6,7,8]. Quantifying plant phenology and interactions with weather and climate is essential in the face of changes in climate and demonstrated shifts in phenology [8,9,10,11]. Repeated years of phenological monitoring can provide information on plant community responses to climate variability, land management activities, and disturbances such as fire and drought. Land-based plant phenology cameras, hereafter referred to as phenocams, have been established at many sites worldwide [12]. Since the inception of plant phenology networks, such as the PhenoCam network [13], an extensive body of literature has been published using standard red, green, and blue (RGB) images from stationary land-based cameras. RGB images and their extracted digital numbers (DN) have successfully been used to calculate various indices of greenness and track plant phenology [1,3,12]. These indices have been shown to be particularly well correlated to phenology in deciduous forest systems [6,14,15,16]. Recent studies from semi-arid regions have demonstrated the utility of land-based cameras for more xeric systems [17,18,19]. However, there are limited studies testing the use of land-based cameras for tracking plant phenology in snow dominated semi-arid regions, such as the Great Basin.

Initially phenocams mainly relied on RGB images to determine indices of “greenness” [1,6]. However, recent advances in both cameras and the development of new indices [20,21], has provided the impetus to examine the fidelity of these approaches using near-infrared (NIR) reflectance images in conjunction with RGB images. Phenocams can be equipped with a NIR enabled-filter which increases the variety of indices that can be derived from the images. If phenocams are equipped with a NIR enabled-filter, sets of RGB and NIR images taken at nearly the same time (i.e., just a few minutes apart) can be used to compute the Normalized Vegetation Difference Index (NDVI). NDVI represents the physiological activity of vegetation; since NDVI relies on the NIR and red reflectance, which are related to the amount of mesophyll tissue and chlorophyll needed for photosynthesis in leaves, respectively [22]. NDVI has been shown to be an effective index for monitoring plant vigor from ground and satellite-based imaging platforms in the Great Basin [23,24,25]. However, an in-depth assessment of the utility of land-based phenocams to monitor plant phenology with NDVI and their agreement with satellite-derived NDVI has not, to our knowledge, been explored in this region. Though one Great Basin shrub site from a field site in Burns, OR, USA has been included in two global studies looking at the utility of phenocams data across multiple biomes [12,26].

Phenocams can provide species-specific information but at a limited number of sites. Therefore, large area coverage is only possible through the use of satellite-based sensors, spurring recent efforts to validate satellite-derived phenology with phenocam-derived phenology. Recent studies found good agreement between MODIS (Moderate Resolution Imaging Spectroradiometer) and phenocam derived non-NDVI based indices of plant greenness. A common index of greenness from phenocam RGB images is the Green Chromatic Coordinate (GCC) [14,19,26,27,28,29,30,31]. In these studies, MODIS data were primarily used to compute NDVI and the Enhanced Vegetation Index (EVI), while phenocams were used to calculate several indices based on the RGB digital numbers. The next development focused on the use of NIR in conjunction with RGB images to examine NDVI and their agreement satellite-derived NDVI [26,29,32].

While satellite based NDVI has been well published, there is far less literature comparing phenocam NDVI to satellite-derived NDVI, since it is an emerging measurement technique [26,29,32]. Petach et al. [32] used a laboratory experiment to test the ability of phenocams to characterize red and NIR reflectance, compute NDVI, and compare it to NDVI derived from radiometric measurements. They found it was necessary to develop correction regressions using measurements from a spectroradiometer. They subsequently used field-acquired StarDot phenocam images from a temperate deciduous forest and found a strong agreement between MODIS derived NDVI and phenocam NDVI, thereby paving the way for others to test these relationships in other vegetation types [26,29]. Work by Liu et al. [29] reviewed the specifics of different satellite sensors: Advanced Very High Resolution Radiometer (AVHRR), Moderate Resolution Imaging Spectroradiometer (MODIS), Visible Infrared Imaging Radiometer Suite (VIIRS), and higher spatial resolution at decreased temporal resolution Landsat data, and compared NDVI from these satellite platforms to phenocam NDVI. They found good correlation between satellite and phenocam NDVI for two oak/grass savanna sites in California. However, the determination of phenophase dates varied between phenocams and satellite-derived NDVI due to different view angles and spatial resolution, with homogenous grassland terrain being more consistent across the various platform, in comparison to mixed oak/grass savannas. Phenophase dates, also referred to as threshold dates, are dates that correspond to an observable stage in the life cycle of plant, such as spring green-up [33] In another study in mixed oak-grass ecosystems, Luo et al. [21] used four sites in Europe to determine the efficacy of different vegetation indices, although they made no comparisons to satellites platforms. Luo et al. [21] found phenocam NDVI was best at characterizing fall senescence, while a new index of NIR reflectance [20] was better during spring green-up. Filippa et al. [26] across 17 sites comprising six different plant functional types compared MODIS NDVI to phenocam NDVI and GCC. The seasonality of phenocam NDVI was comparable to both ground spectral sensors and MODIS. Additionally, phenocam NDVI remained higher during fall senescence relative to phenocam GCC. They attributed this to phenocam GCC being more sensitive to changes in leaf color, while phenocam NDVI appeared more sensitive to structural changes, such as changes in leaf area. Based on prior research, a logical next step is to determine the agreement between phenocam-based NDVI and satellite-based NDVI in other vegetation communities. Comparing these two approaches may provide insight on the causes of potential differences and limitations of each approach.

Satellite-derived indices vary on scales of meters to kilometers, and the pixels are representative of a mixture of vegetation types and ground area (i.e., mixed pixels) [20]. MODIS NIR and red reflectance data is at a relatively coarse spatial resolution (250 m × 250 m) and acquired daily (~four days at near nadir view angles), whereas Landsat has finer spatial resolution (30 m × 30 m) but is only acquired every 16 days. In systems with heterogeneous vegetation, finer spatial resolution is necessary for tracking the phenology of distinct community types, and those that are relevant to management. For example, the Great Basin is characterized by north-south trending mountain ranges interspersed with flat, aggraded valleys. Groundwater dependent systems, including meadows and riparian stream corridors, occupy a small fraction of this semi-arid landscape, yet are vitally important sources of water and habitat to sustain biodiversity of plants and animals [34,35]. Mountain ranges in the area often have dramatic elevation gradients and contain steep, narrow canyons, leading to heterogeneous vegetation. Given the need for relatively high spatial resolution to track phenology of distinct vegetation types, we relied on Landsat since it has sensor continuity and adequate spatial and temporal resolution, to explore the amount of agreement between Landsat-based NDVI and phenocam-based NDVI. Leveraging the multi-decadal record of Landsat NDVI, while combining these data with increased spatial and temporal resolution phenocam NDVI can potentially aid in enhanced understanding of plant phenology dynamics at finer scales in the study area and elsewhere.

There is little research comparing Landsat to phenocam NDVI, especially in the Great Basin and other regions where groundwater dependent ecosystems are prevalent. This study used StarDot web cameras and camera data processing methods standardized by the PhenoCam Network [33,36]. The objectives of this study are to (1) quantify the level of agreement between phenocam NDVI and Landsat NDVI in five community types characteristic of the Great Basin, (2) quantify the agreement between NDVI-based phenophase dates, and (3) study the effect of observation year and species composition on phenology metrics for three years with variable weather spanning drought to wet conditions in sagebrush steppe, meadow, and pinyon and juniper vegetation. Results from this work will help to assess the utility of these two approaches for monitoring patterns of phenology in these community types and gauge if phenocams can bridge the temporal gaps of Landsat.

## 2. Materials and Methods

### 2.1. Study Area

**Figure 1:**
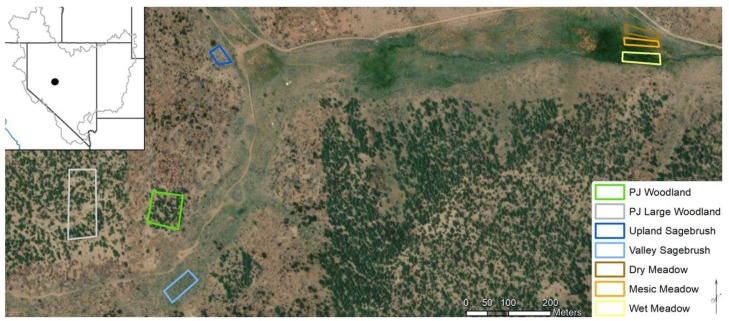
Map showing the location of Porter Canyon Experimental Watershed (PCEW) located in the Desatoya Mountain Range in Central Nevada, USA, gray outline on the inset is the boundary of the Great Basin. The location and size of the seven phenocam regions of interest (ROIs) used to determine the relationship between phenocam NDVI and Landsat NDVI are shown on Google, Digital Globe imagery of PCEW. Pinyon and juniper woodlands are denoted as PJ woodlands on the figure legend.

Three years of data from multiple phenology cameras were obtained within the Porter Canyon Experimental Watershed (PCEW) located in the Desatoya Mountains of Central Nevada (39°28′ N; 117°37′ W, 2195 m elevation), which is part of the Great Basin (Figure 1). The Great Basin is located in the western United States and includes most of Nevada (Figure 1). Climate is characterized by cold, wet winters and warm, dry summers for this cold desert region. The long-term average precipitation for the study area is 360 mm with approximately 65% of the precipitation in winter months, mainly as snow [37]. The monthly average temperature ranges from a minimum of −11.5 °C in January to a maximum of 27.8 °C in August [37]. For a more detailed description of the study area see Snyder et al. [18].

Vegetation is comprised of three main community types: singleleaf pinyon (*Pinus monophylla*) and Utah juniper (*Juniperus osteosperma*) woodlands, mountain big sagebrush (*Artemisia tridentata* ssp. *tridentata*) shrublands, and high elevation valley bottom meadows. The diverse meadow community was differentiated into three classes based on vegetation type for analysis. The wet meadow consists of Nebraska sedge (*Carex nebrascensis*) and arctic rush (*Juncus arcticus);* the mesic meadow has a diverse mix of forbs, arctic rush, and Douglas’ sedge (*Carex douglasii*). The dry meadow currently has an overstory of mountain and basin big sagebrush (*Artemisia tridentata* ssp. *tridentata)* with primarily Douglas’ sedge and povertyweed (*Iva axillaris)* in the understory. Pinyon and juniper (PJ) are native coniferous evergreen trees. Sagebrush is considered a semi-deciduous evergreen shrub as it retains leaves throughout the year. Vegetation surveys were conducted in 2017 to characterize the plant communities using standard line point intercept methods [38]. The interspace in the pinyon and juniper community had a mixture of bareground (62.6%) and basal cover of forbs and grasses (37.4%), dominant grasses were cheatgrass (*Bromus tectorum*) and Sandberg’s bluegrass (*Poa secunda*). Both cheatgrass and Sandberg’s bluegrass are early season grasses with cheatgrass being an annual and Sandberg’s blue grass being perennial. Sagebrush has two sets of leaves: a persistent set that grows in spring and summer and are retained through winter; and an ephemeral set of larger leaves that emerge in spring, but are senesced at the height of the summer dry period with the previous year’s persistent leaves [39]. Artic rush, Nebraska sedge and Douglas’ sedge are perennial species with varying phenologies [40]. Artic rush and Nebraska sedge flower from late May to August with seed ripe typically occurring in August, whereas Douglas’ sedge flowers in May to late June [41]. Povertyweed, a rhizomatous, perennial herb exhibits a wide flowering window. The plant’s bloom period occurs between April and October, primarily in response to moisture availability [41]. 

**Figure 2:**
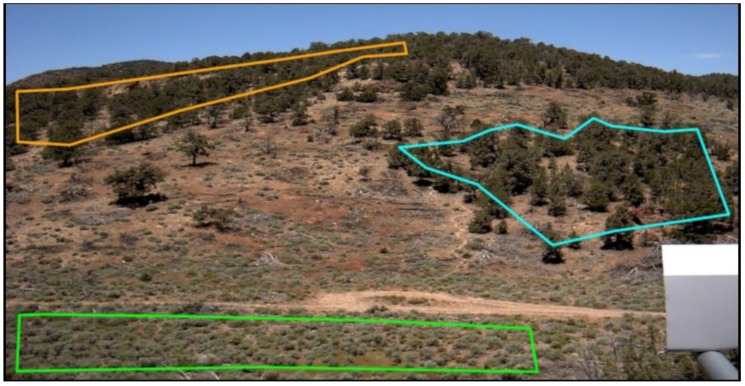
Phenocam regions of interest (ROIs) for the pinyon and juniper woodland & valley sagebrush camera site. The light blue ROI is the small pinyon and juniper woodland site that was scaled for fractional cover. The orange ROI is the larger pinyon and juniper woodland ROI used to check results for the smaller pinyon and juniper woodland site. The green ROI is the valley sagebrush site.

**Figure 3:**
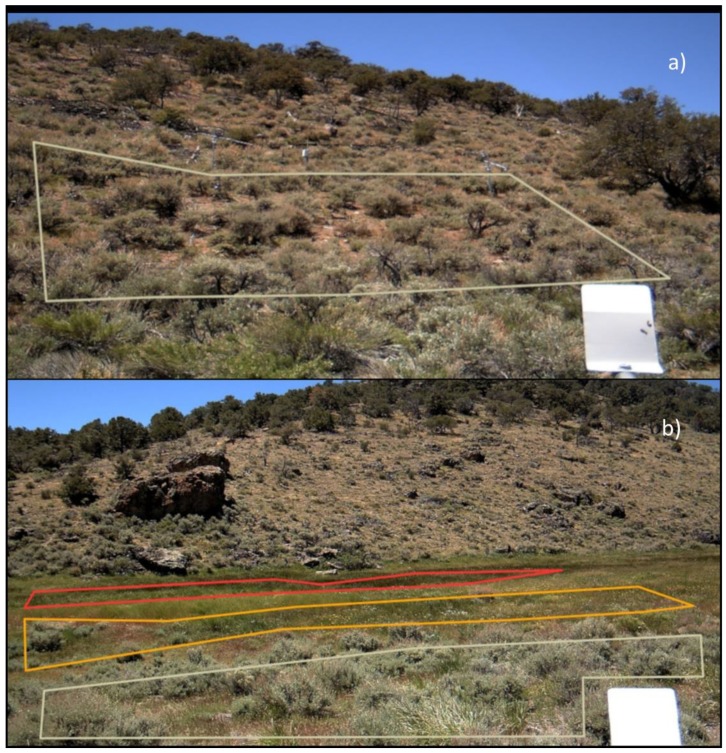
(**Panel a**) is the region of interest (ROI) for the upland sagebrush camera site, upland sagebrush is outlined in white. (**Panel b**) shows the three ROIs for the meadow camera site: wet meadow (red ROI), mesic meadow (orange ROI), and dry meadow (white ROI). The dry meadow has experienced historic degradation and has a sagebrush component.

Three phenocams were installed to obtain the land-based imagery and estimates of NDVI for each of the community types in the regions of interest (ROIs) from 2015 through 2017 (Table 1, Figure 2 and Figure 3). The following six ROIs were defined and analyzed: a small PJ woodland site located in the middle of a cutting treatment, sagebrush located on the valley floor (i.e., valley sagebrush), upland sagebrush, wet meadow, mesic meadow and dry meadow (Figure 1, Figure 2 and Figure 3). A seventh ROI was defined to confirm results from the small PJ ROI, this was a larger PJ woodland located upslope of the small PJ ROI (Figure 1 and Figure 2). The description of the three cameras is provided, and includes heights of the cameras, angle mounted from horizontal to the ground surface, azimuth and aspect (Table 1). Commonly used abbreviations are provided in the Supplementary Information (Table S1). 

### 2.2. Climate Data

Micrometeorological variables were measured at a Natural Resources and Conservation Service SNOTEL station located within the study area [42]. Air temperature and relative humidity were used to compute the daily vapor pressure deficit (VPD) as the difference between the average daily e_s_ and e_a_, where e_s_ is the daily average saturation vapor pressure at air temperature, and e_a_ is the daily average actual vapor pressure. Daily precipitation was used to compute water year totals (1 October–31 September) for the each of the study years.

### 2.3. Phenology Cameras

The study used Star Dot web cameras and camera data processing methods standardized by the PhenoCam Network Website [33,36]. Phenocam images were acquired by StarDot NetCam SC 5MP IR-enabled cameras using a complementary metal oxide semiconductor (CMOS) image sensor at a resolution of 1296 × 960 pixels and a lens focal length of 6.2 mm. The cameras take a colored three-layer Red, Green, Blue (RGB) image and a RGB + Near Infrared (NIR) image. These sets of images were taken 30 s apart to allow for the movement of the camera’s mechanical filter.

### 2.4. Phenocam Acquistion, Filtering & Processing

In 2015, 6 sets of RGB and RGB + NIR images were taken per day every two hours between 8 a.m. and 6 p.m. However, this caused problems due to varying camera orientations and light conditions, consequently many images had to be discarded. Therefore, after 2015 in all subsequent years, the sampling window for acquiring images was changed to a two- and half-hour window around the time of day that produced the highest quality images which were less likely to be affected by solar glare and shadows. The time of day most suitable for image acquisition varied with camera orientation, but 6 sets of RGB and RGB + NIR images were taken in two- and half-hour window every 30 minutes between 10:00 a.m. and 2 p.m. This greatly reduced the number of images with poor exposure, high noise, offset white balance, lens flare, and high contrasting shadows. For example, in 2015 the number of images removed were approximately 24% of PJ woodland & sagebrush valley camera, 21% of the images from upland sagebrush, and 7% of the meadow images; in 2016, the number of discarded images dropped to 7, 8 and 0.5%, respectively.

The user-defined ROIS (Figure 2 and Figure 3) on the images were processed using the *Phenopix* package available in the R programming environment [33]. Phenocam-derived NDVI was computed within *Phenopix* following Liu et al. [29] and Petach et al. [32]. NDVI was calculated as Equation (1) from Petach et al. [32]:

(1) $$NDVI=\;\frac{\left(NIR-Red\right)}{\left(NIR+Red\right)}$$

where *NIR* is the near infrared and *Red* is the red, adjusted digital numbers. Prior to computing NDVI, several image pre-processing steps were performed and are described below. Image exposure values were extracted using *Phenopix*. To adjust the pairs of images at each timestep, the digital numbers (DN) extracted from the images are adjusted by exposure time using Equation (2)

(2) $$\;DN_{A}=\;DN/\sqrt{E}$$

where *DN_A_* is the adjusted DN values and *E* is the exposure time. For details on the exposure adjustment for the StarDot phenocams see Petach et al. [32]. Data were filtered for extreme values using the exposure data. RGB + NIR images had consistently lower exposure values on StarDot cameras than RGB images. Therefore, if an RGB image had a lower exposure value than the corresponding RGB + NIR image, this set of images was removed. Additionally, exposure values above 1600 were removed due to significant degradation in raw image quality.

The DN values of NIR component are extracted from the adjusted RGB + NIR DN values using a gray-scale equation Equation (3) from Petach et al. [32]:

(3) $$DN_{NIR\_A}\;=\;DN_{\left(RGB+NIR\right)\_A}-\left(0.30*\;DN_{R\_A}+0.59*\;DN_{G\_A}+0.11*\;DN_{B\_A}\right)$$

where *DN_NIR_A_* is the adjusted NIR DN value, *DN*_(*RGB+NIR*)*_A*_ is the adjusted RGB + NIR DN, and *DN_R_A_*, *DN_G_A_* and *DN_B_A_* are the adjusted red, green, and blue DN values, respectively. Subsequently, phenocam NDVI was calculated using Equation (1). Phenocams provide digital numbers (DN) not actual reflectance values, and therefore the majority of values are negative for phenocam NDVI [29]. Petach et al. [32] provided linear rescaling methods to account for this, however these linear re-scaling methods do not affect the seasonal cycles and therefore similar to Liu et al. [29], it is not implemented here. However, values were rescaled for graphical purposes, as discussed below.

Images containing snow were removed from the analysis. Images containing snow were associated with relatively high percentages of blue (blue chromatic coordinate), therefore we used a cumulative distribution to identify the different linear slopes. Each site’s blue chromatic coordinates (BCC) were ordered into a cumulative distribution and used in the *breakpoint* function of R’s *strucchange* package, and values greater than the BCC breakpoint were removed since these images contained snow. Further details can be found in Snyder et al. [18] where a similar approach was applied to green chromatic coordinates (GCC) instead of BCC.

The remaining sub-daily data were further filtered and smoothed using *Phenopix*’s *autoFilter* function. The applied autofilters were *blue*, *spline*, & *max*. The *blue* filter found the 90th percentile of each day’s standard deviation and removed any data that was twice its distance from the mean. This removed extreme outliers and swings in the sub-daily data. The *spline* filter followed methods described in Migliavacca et al. [43] and was used with an “up/down” parameter value of 1.6. This parameter value caused the spline filter to be slightly more stringent than the default values in the *Phenopix* package, however phenocam NDVI data required more smoothing than the greenness chromatic coordinate (GCC) which uses only the RGB image (see details in Snyder et al. [18]). The *max* filter followed Sonnentag et al. [3] methods and removed points outside the 90th percentile of a three day rolling window of sub-daily NDVI data. After filtering, sub-daily values were then aggregated to daily median. 

### 2.5. Landsat Data Processing

Landsat 8 Optical Land Imager (OLI) satellite images were used to compute NDVI from 2015 through 2017, according to Equation (1), where *NIR* is the near infrared at-surface reflectance, and *Red* is the red at-surface reflectance. Landsat Tier 1 Surface Reflectance data were accessed via Google Earth Engine, who produced the Surface Reflectance collection following USGS [44,45] via a Docker image supplied by the USGS. The process converts Landsat OLI top-of-atmosphere reflectance to at-surface reflectance using the Landsat Surface Reflectance Code (LaSRC) atmospheric correction algorithms [45]. Landsat Surface Reflectance data were used to compute NDVI for cloud free pixels. Clouds were identified and masked using the cloud mask band derived from CFMask [46].

### 2.6. Spatial Averaging of Regions of Interest

Regions of interest (ROI) were manually defined that maximized the area of the target vegetation community (Figure 2 and Figure 3). Phenocam ROIs ranged in width and length from 100 m × 200 m to 40 m × 46 m (Figure 1). Landsat NDVI pixels coincident with each ROI were identified and selected (ranging from two to three pixels) and were spatially averaged for each image. Phenocam NDVI images were spatially averaged for each ROI using all pixels, except for the pinyon-juniper ROI. These spatially averaged ROI NDVI datasets were used in the phenology analysis described below (Section 2.7). For the pinyon and juniper woodland ROI a slightly different approach was used; it became apparent that the interspace and canopy had different patterns, so we analyzed canopy cover separate from interspace pixels (Figure 2), similar to Liu et al. [29]. An unsupervised clustering function in *Phenopix* was used to identify two cover types, pinyon and juniper canopy and interspace on the phenocam images using the three-year dataset of images. Interspace areas were a combination of understory vegetation, bare ground, litter, woody material, and shadows. For cluster analysis, each pixel was identified by its 3-year quantiles from 2.5% to 97.5% by 1% steps and its standard deviation. Pixels were classified as either canopy cover or interspace. The two classes of pixels were averaged together by median and then treated like an average ROI for the remaining analyses. To scale back up for comparison with Landsat, we used the fractional cover of canopy and interspace derived from the National Agricultural Imagery Program (NAIP) imagery. This was done to make the more oblique phenocam view more comparable to the nadir view of Landsat. This was only done for pinyon and juniper ROI because the vegetation height obscured some of the interspace area, and was not necessary in the shorter statured sagebrush and meadow communities

To re-scale the data for comparison against a 30 m × 30 m Landsat pixel, we used a supervised classification on NAIP imagery to determine the percentage of pinyon-juniper and interspace. The supervised classification was performed on a single NAIP image of the study site. We used the *nnet* package in R and 100 training points were used and were classified with 98% accuracy (see Supplementary Information, Figure S1). Lastly, canopy and interspace NDVI values were scaled by their percent cover into one composite number using Equation (4) [29]:

(4) $$NDVI_{P\_S}=F_{can}*NDVI_{P\_can}+\;F_{int}*NDVI_{P\_int}$$

where *F_can_* and *F_int_* represent canopy and interspace fractions, *NDVI_P_can_* and *NDVI_P_int_* are the phenocam NDVI of canopy and interspace, *NDVI_P_S_* is the phenocam composite NDVI that is scaled up to the Landsat spatial scale using NAIP imagery. The scaled up composite NDVI was then used as described below, for fitting seasonal curves and determining phenophase dates. This was only implemented for the small pinyon and juniper woodland ROI (shown in light blue, Figure 2), the larger pinyon and juniper ROI (orange outline, Figure 2) was only used to confirm the general average ROI trends in NDVI.

### 2.7. Phenology Analysis

Annual phenocam and Landsat NDVI datasets were processed using *Phenopix* functions to assess phenology patterns for each year. The thresholds represent the estimated dates of important phenophases in plant development. Start of Season (SOS) are the range of dates associated with noticeable green-up of the plant community in the imagery. Peak of production (POP) are the dates associated with height of plant vigor and corresponds to peak production. End of season (EOS) are the dates when plants begin senescence, chlorophyll content declines, and plants become less vigorous and green. To determine these threshold date ranges, each year was processed using the default *spline* fitting and a modified *trs* thresholding method at 5000 replications for the uncertainty analysis. The uncertainty analysis is a measure of how well the fitted curve and extracted threshold dates fit the data. The residuals between the fitted and observed values were used to generate random-noise in the data, and fitting was applied recursively to randomly-noised data. Phenophase dates were determined with an associated uncertainty estimate.

Important phenophase dates were determined with a modified *trs* method. We modified the *trs* thresholding methods for increased seasonal resolution. A basic assumption of the unmodified trs thresholding is that the annual minimum is nearly the same each year [33]. However, perennial communities, or communities with winter vegetation can have different annual baselines. *Phenopix*’s default thresholding for spline fitting is “*trs*” at a value of 0.5. This finds the 50% value of all positive-slope and negative-slope data, to assign SOS and EOS dates, respectively. Our modified version split the annual data by its POP value. Subsequently, SOS and EOS are chosen using slope data pertaining to either the increasing or decreasing part of the seasonal NDVI curve. To compare phenocam and Landsat thresholds, we produced *trs* thresholds at 0.15, 0.25, 0.35, 0.5, 0.65, 0.75, and 0.85 of the green-up amplitude and did the same on the downward side of the seasonal curve for EOS. Though our threshold method is slightly altered from White et al. [47], for all tables we used the standard 0.5 value with 95% CI. Just as White et al. [47] concluded, this 0.5 threshold value turned out to be the most representative and stable date range when comparing across sites, years, and between platforms. Length of Season (LOS) is defined as EOS minus SOS.

For visual comparison purposes, data used in the figures were rescaled from zero to one across the three years. Variations between years and Landsat versus phenocam are highlighted with this method. To rescale figure data, we took the final NDVI data for each site from both phenocam and Landsat, determined the 3-year minimum, maximum and amplitude, then subtracted the minimum from each data point and divided by amplitude. Values were rescaled for both Landsat and phenocams using Equation (5).

(5) $$NDVI_{Re}=\frac{NDVI_{orig}-NDVI_{min}}{NDVI_{max}-NDVI_{min}}$$

*NDVI_Re_* is the re-scaled NDVI for either Landsat or phenocam, *NDVI_orig_* is the original value, *NDVI_min_* is the minimum value of the three-year dataset, and *NDVI_max_* is the maximum value of the three-year dataset divided by the three-year amplitude (amplitude = *NDVI_max_* − *NDVI_min_*). These scaled values were used in the figures that show the seasonal trends of NDVI for each ROI.

Root mean square errors (RMSE) were calculated using the rescaled data to compare total differences in variation between Phenocam and Landsat on a pairwise basis. For each data point of Landsat, the associated day of phenocam data were compared. Per year RMSE and three-year average RMSE were calculated and shown in Table 2. Pearson’s *r* correlation coefficients were also calculated on these data and shown in Table 3. However, the values in Table 4 (minimum, maximum and amplitude) are the un-scaled actual NDVI values from phenocams and Landsat. Furthermore, values presented in Table 2, Table 3 and Table 4, and Supplemental Information (Table S2), labelled as “pinyon & juniper woodland” are from the smaller pinyon and juniper ROI (shown in light blue, Figure 2). The relationship phenocam and Landsat phenophase dates were plotted relative to a 1:1 line. The difference between phenocam and Landsat dates was then calculated. The absolute difference between dates was used to calculate a student *t*-test to determine if the difference varied significantly from zero for each phenophase: SOS, POP, and EOS. The average absolute difference and standard deviation were calculated to determine the dispersion from a 1:1 relationship.

## 3. Results

### 3.1. Climate

**Figure 4:**
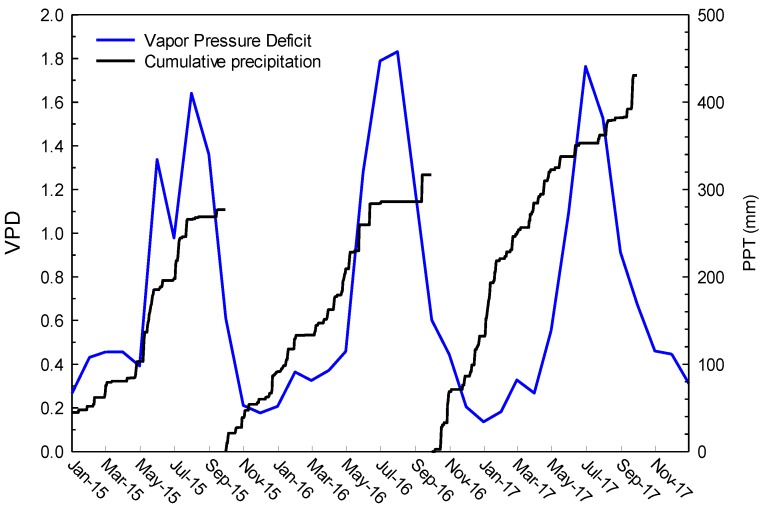
Average monthly vapor pressure deficit (VPD, blue line) and cumulative precipitation (PPT, black line) for the water years 2015 to 2017.

Water year 2015 was the fourth year of a continuous drought with below long-term average precipitation (276.6 mm), 2016 was slightly below average long-term precipitation (316.7 mm), and 2017 was above average precipitation (430 mm). In terms of percent of average annual precipitation, the water-years 2015–2017 were 76, 88, and 119% of average long-term precipitation, respectively. VPD was near average each year, being lower in spring and fall and increasing in the summer (Figure 4).

### 3.2. Pinyon and Juniper Community

**Figure 5:**
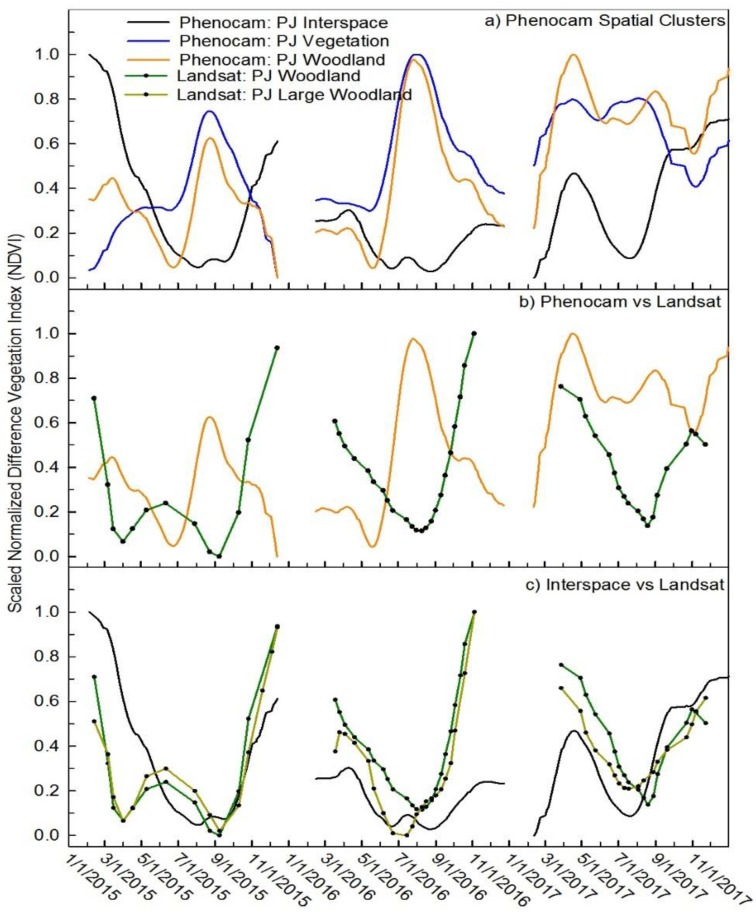
Scaled NDVI values comparing seasonal trends from phenocam and Landsat. (**Panel a**) Phenocam NDVI from the pinyon and juniper (PJ) woodland ROI, the blue line is the PJ canopy, the black line is the interspace, and the orange line is the scaled-up NDVI that resulted from scaling the NDVI values of the PJ canopy and the interspace by their fractional cover. (**Panel b**) Landsat NDVI (green) from Landsat pixels coincident with the PJ woodland ROI versus phenocam woodland NDVI (orange). (**Panel c**) Landsat NDVI of the PJ woodland (green), the phenocam NDVI of the interspace (black), and Landsat NDVI from the larger ROI of PJ woodland (olive) located uphill from the primary study site, see the gray box in Figure 1.

The NAIP classification resulted in 62.8% canopy cover and 37.2% interspace area. The interspace area-averaged ROI demonstrates an opposite seasonal trend in phenocam NDVI than the canopy area-averaged ROI, where the interspace phenocam NDVI is highest in the spring and fall and lowest in the summer (Figure 5a). This is opposite to the green-up signal from the canopy during the growing season. When phenocam NDVI values were averaged by fractional cover of interspace and canopy, NDVI values declined between May and June. This decline in NDVI could be due to the changes in soil reflectance because of soils drying-down during the growing season or to early senescence of the dominant early season grasses in the interspace (Figure 5a, orange line). The wet year of 2017 is the notable exception with soils apparently being wetter later into the month of May and then drying-down. In addition, the canopy NDVI signal did not show a clear green-up response in 2017 because the NDVI was already high at the beginning of the season.

The phenocam NDVI is compared to Landsat NDVI in Figure 5b. Results indicate that the Landsat NDVI is influenced by the interspace signal and is thus opposite of the scaled phenocam NDVI. The lack of fit between the two platforms is reflected in a high RMSE (>0.4) and the inverse relation between the two fits was reflected in the negative Pearson’s correlation (*r ≤* −0.24). The interspace phenocam NDVI was somewhat in agreement and correlated to the Landsat (Table 2 and Table 3). However, a different pattern occurred in 2015, the phenocam interspace NDVI declined yet Landsat NDVI increased from April through July. The RMSE between the Landsat and phenocam interspace was somewhat improved and averaged 0.26. The agreement between the phenocam interspace and Landsat was reflected in a positive, but weak correlation (*r* ≤ 0.44). Because the pinyon and juniper woodland site is located in the middle of a harvested area and is relatively small (75 m × 75 m), we explored if Landsat results might be affected be a geo-rectification problem, and unduly influenced by the surrounding harvested areas. Therefore, we selected three adjacent Landsat pixels in the woodland above the study site to determine if geo-rectification problems were contributing to the seasonal trends (area is shown in Figure 1 and Figure 2). Results from this analysis confirmed that the original seasonal trend from the study site was also present in the three Landsat pixel average and compared well to the interspace phenocam NDVI (Average all year RMSE = 0.05, and *r* = 0.17) (Figure 5c), indicating that the pattern is not a result of geo-rectification problems and that Landsat NDVI signals are similar in the both pinyon and juniper ROIs. This could be due to changes in soil reflectance and/or due to senescence of the dominant early season grasses in the interspace as discussed below. As mentioned in the methods, values presented in Table 2, Table 3 and Table 4 labelled as “pinyon & juniper woodland” are from the smaller pinyon and juniper ROI (shown in light blue, Figure 2).

### 3.3. Sagebrush Communities

**Figure 6:**
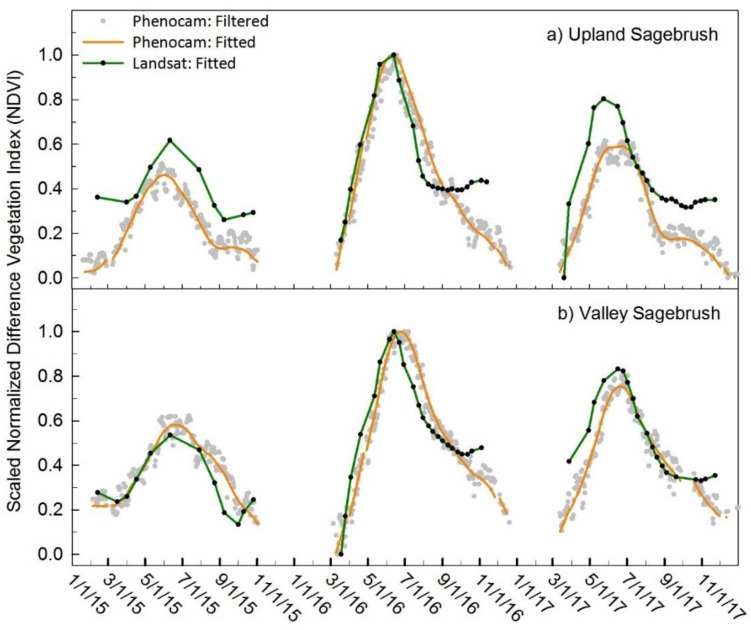
Scaled NDVI values are presented to compare the seasonal trends of phenocam and Landsat. Phenocam NDVI (orange) and Landsat NDVI (green) of the sagebrush communities at (**panel a**) upland sagebrush site and (**panel b**) the valley sagebrush site.

Phenocam and Landsat NDVI comparisons for the sagebrush communities are illustrated in Figure 6, indicating that the two datasets compare well, particularly during the green-up period and at the peak of season. The discrepancy between the two datasets is most pronounced from late August through the fall when phenocam NDVI declines, but Landsat NDVI stabilizes and even increases slightly during the late fall. This stabilization and slight increase could be due to yellow flowers of the fall-blooming rabbitbrush, as described below. In 2015 and 2017, there was also a small increase or general flattening of the senescence curve during the fall in the phenocam NDVI. This was the pattern for both the valley sagebrush site and the upland sagebrush site. Visual inspection of the phenocam images revealed that in the spring the understory grass and forbs started greening up first in April, in early May sagebrush greened up, followed by rabbitbrush. In midsummer the understory had turned brown, and sagebrush started to look less green. The small increase in the fall appears to be the blooming of the rabbitbrush which produces yellow pixels in the ROI. Sagebrush was never leafless, but overall had a much more subtle variation in greenness [18]. The upland sagebrush and the valley sagebrush both had lower RMSE values indicating a better fit between the two datasets, with an average across the 3-years of 0.16 and 0.09 at the upland and valley sites, respectively (Table 2). The Pearson’s *r* values were strongly correlated ranging from 0.84 to 0.93 (Table 3).

### 3.4. Meadow Communities

**Figure 7:**
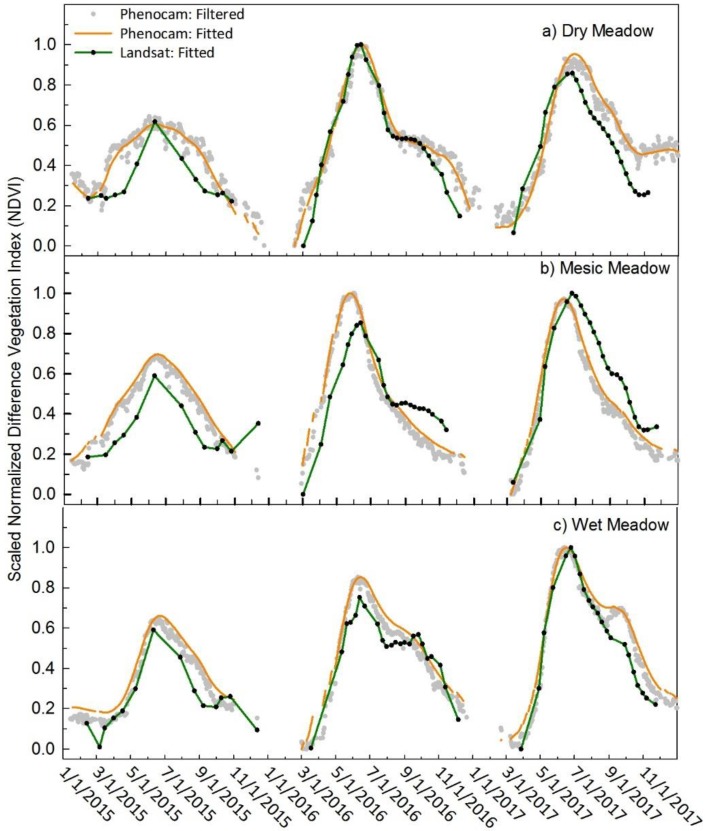
Scaled NDVI values are presented to compare seasonal trends of phenocam and Landsat. Phenocam NDVI (orange) and Landsat NDVI (green) of the sagebrush communities at (**panel a**) dry meadow, (**panel b**) mesic meadow, and (**panel c**) wet meadow.

The agreement between satellite NDVI and phenocam NDVI for all years was similar in the dry meadow sagebrush dominated site as compared to the other two sagebrush sites (Figure 7a). The RMSE was 0.12 averaged across the three years (Table 2). The three years had good agreement, particularly during green-up with more discrepancy during senescence, similar to the other sagebrush sites (Figure 6a and Figure 7a). The mesic meadow showed good agreement between the two datasets with RMSE averaging 0.14 across the three years. The dry year of 2015 had the poorest fit (RMSE 0.14) (Figure 7b, Table 2). The wet meadow had strong agreement between phenocam and Landsat NDVI (Figure 7c, Table 2) and had the most consistent RMSE on a yearly basis, and across all years (RMSE = 0.11). The consistency between years is likely due to high density of vegetation because of shallower groundwater levels, and this community’s clear response to seasonal temperature cues and high degree of greenness and vigor which produce a greater seasonal amplitude in NDVI (Table 4). Pearson’s *r* values showed a high degree of correlation for all three meadow ROIs (Table 3), the lowest correlation was in the dry year of 2015 in the dry and mesic meadow and is primarily driven by the lack of cloud free images in the Landsat dataset, resulting in only 10 to 12 data points.

### 3.5. Comparison of Phenophase Dates

**Figure 8:**
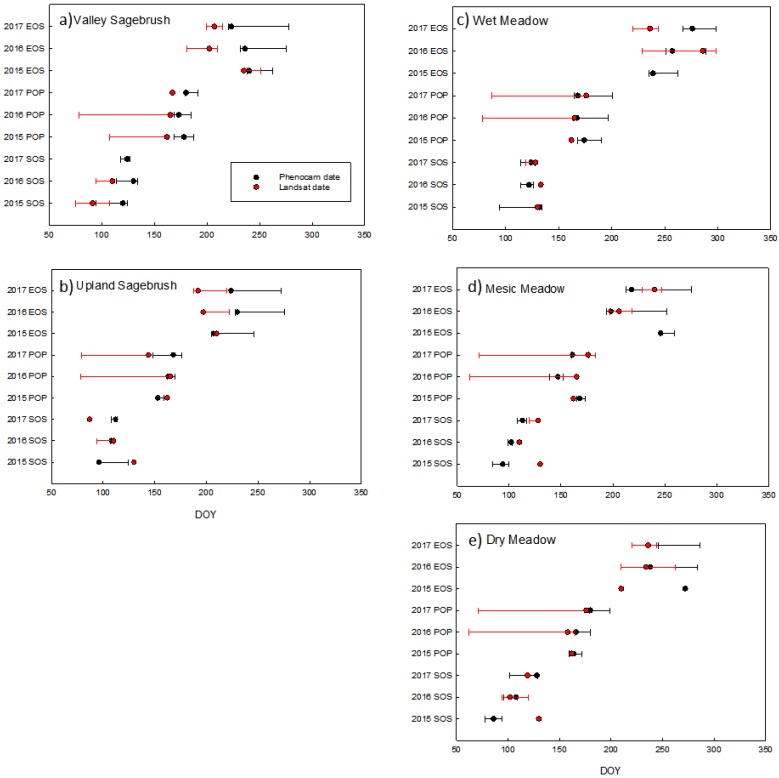
Landsat and phenocam phenophase dates and confidence intervals for: (**a**) Valley Sagebrush, (**b**) Upland Sagebrush, (**c**) Wet Meadow, (**d**) Mesic Meadow and (**e**) Dry Meadow. Phenophase dates are start of season (SOS), peak of production (POP), and end of season (EOS) for the years 2015–2017.

To compare phenology dates of SOS and EOS, we explored using a moving threshold to determine threshold dates at 0.15, 0.25, 0.35, 0.5, 0.65, 0.75, and 0.85 of the upward and downward trending amplitude (data not shown), to determine if this moving threshold would find a percentage of the amplitude that produced the best agreement between phenocam and Landsat SOS dates. There was large variation between years, so we choose to use the standard 0.50 threshold of the amplitude during the green-up and senescence period as proposed by White [47]. In general, thresholds of 0.35 (data not shown) and 0.50 provided reasonable agreement in threshold dates between the Landsat and phenocam NDVI. We plotted the SOS, POP and EOS threshold dates and the associated asymmetrical 95% confidence interval of the fitted threshold dates for the two sagebrush sites and the three meadow sites (Figure 8). However, due to limited Landsat data some thresholds and confidence intervals were not able to be fit, or there was not enough data to generate predictions of confidence intervals (Figure 8). It was not possible to extract and compare phenophase dates for the pinyon and juniper woodland, due to the irregular shapes of the Landsat curves.

**Figure 9:**
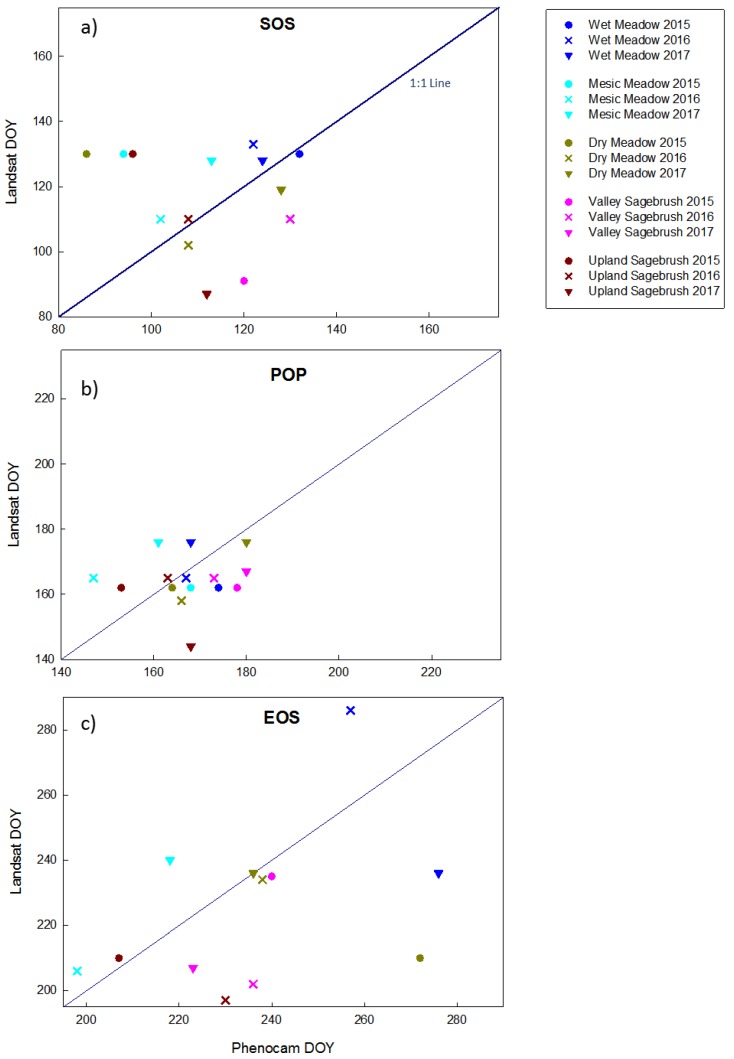
The relationship between Landsat and phenocam phenophase dates plotted relative to a 1:1 line. (**Panel a**) start of season (SOS), (**panel b**) peak of production (POP), and (**panel c**) end of season (EOS). Colors represent sites, which were ordered from wet to driest, and symbols represent years. Each graph panel is scaled to encompass 95 days; thus graphs can be directly compared.

SOS and POP dates agreed reasonably well at the two sagebrush sites as seen by the degree of overlap. For the valley sagebrush, Landsat dates were consistently earlier than phenocam dates. In the upland sagebrush Landsat dates were generally earlier as well, except for SOS in the dry year of 2015, when Landsat SOS was later than phenocam SOS, however in this dry year the best EOS agreement was seen in both upland and valley sagebrush (Figure 8). In general, the three meadow communities had more overlap in extracted phenophases dates, but there were some notable discrepancies (Figure 8). In the mesic meadow there was a consistent trend that Landsat predicted later dates than phenocam (Figure 8). To quantify how Landsat dates varied from phenocam dates we plotted the relationship in relation to a 1:1 Line (Figure 9), the sites were color coded and ordered along an aridity gradient from wetter to drier. A student *t*-test on the absolute difference determined that the date difference for each phenophase SOS, POP, and EOS, were significantly different from zero (*p* = 0.004, 0.001, and 0.009, respectively). Because there was no discernible trend of Landsat consistently producing earlier or later phenophase dates in comparisons to phenocams, it was necessary to use the absolute difference of the deviation from the 1:1 line. The mean absolute difference between the two platforms was least for POP (10 days ± 6.5 SD) and greater for SOS and EOS (17 days ± 13.9 SD and 18.8 days ± 14.5 SD, respectively). RMSE calculated assuming a 1:1 relationship with phenocam phenophase dates were 21.8, 11.6 and 28.3 days for SOS, POP, and EOS, respectively. SOS in the dry year 2015 showed considerable variation with Landsat predicting a much later SOS in the dry, mesic, and upland sites (Figure 9). POP showed more agreement between platforms, Landsat produced similar POP dates regardless of community type, which is attributable to the low temporal resolution of the imagery with POP being around the second week of June in the dry and average precipitation years and slightly later in June during the wet year. EOS dates showed the most discrepancies between the platforms. The wet meadow had the greatest agreement between platforms for both SOS and POP. Length of season (LOS) did not show any consistent patterns with in or across sites, with the exception that Landsat phenophase dates produced a consistently shorter growing season for the upland sagebrush site (Supplementary Information, Table S2).

### 3.6. Seasonal Amplitude

The seasonal amplitude increased in all community types during 2016 when the drought ended. Interestingly, most communities showed the greatest amplitude in NDVI in 2016 and less in 2017 after the very wet winter; the exceptions were the mesic and wet meadow which had increasing amplitude throughout the three years (Table 4). These differences may be a result of the evergreen shrubs and trees showing a pronounced response to release from drought in 2016, while the wet and mesic meadows were continuing to respond to progressively increasing groundwater levels over the 2016 to 2017 period (T.K. Stringham unpublished data).

## 4. Discussion and Conclusions

Comparisons between phenocam and Landsat based NDVI compared well across all sites with the exception of the pinyon and juniper site. Landsat and Phenocam NDVI were strongly correlated meadow and sagebrush sites (*r ≥* 0.76, RMSE *≤* 0.18). Pinyon and juniper satellite and phenocam NDVI were negatively correlated and did not agree well (*r* ≤ −0.25, RMSE ≥ 0.41). NAIP imagery (1 m resolution) was used to determine the fractional coverage of pinyon and juniper versus interspace areas which were a combination of grasses and forb (37.4% basal cover of vegetation (T.K. Stringham unpublished data)) and bareground, to build a scaling relationship with Landsat 8 data [29]. The interspace signal dominated the shape of the Landsat curve for this community type. A comparison with several other pinyon and juniper Landsat pixels revealed this was not unique to our study site. Our study site and the larger wooded areas were on a steep hillslope (22° slope), which may explain some of the differences between the oblique phenocam angle and the near-nadir view of Landsat. Although we scaled up our phenocam using estimates of cover from the nadir view NAIP imagery, the oblique view of the phenocam through the canopy cover of the trees may have been less influenced by the interspace. The taller statured woodland ROI may have been more influenced by shadows, sun angle, view angle, and terrain angle. It is also possible that these differences in viewing angles in taller–statured vegetation, made the nadir view of Landsat Red and NIR bands more influenced by soil moisture in the interspaces. However, a preliminary analysis of soil moisture 20 cm below the soil surface (data not shown), showed there are complex relationships between interspace NDVI, soil moisture, and canopy NDVI. Alternatively, it seems plausible that the interspace areas that were dominated by cheatgrass and Sandberg’s bluegrass, both cool, early season grasses contributed more to the Landsat NDVI signal, than the relatively low amplitude NDVI signal from the evergreen pinyon and juniper. Cheatgrass and Sandberg’s bluegrass emerge early in the growing season and have typically senesced in June. Cheatgrass is particularly red when senesced which would reduce the NDVI signal.

Other studies in evergreen forest systems have similar results to this study [26,48]. Wingate et al. [48] in several needleleaf forests in Europe, found it was difficult to automatically determine the start of the season for conifers because of snow or camera placement, and the detection of green-up and senescence were subtle changes in the green fraction (GCC) of the canopy. Filippa et al. [26] found a poor relationship between MODIS NDVI and phenocam NDVI and GCC for two evergreen needle forest sites located in Oregon and California. Furthermore, the phenocam index GCC seemed to track green-up of the canopy better, while phenocam NDVI was more influenced by new needle formation and cone development. This is similar to results in Snyder et al. [18], phenocam GCC of individual tree canopies were averaged and able to detect subtle changes in seasonal canopy greenness, while NDVI of the tree canopy in the current study was more variable and likely was influenced by needle development. Further investigation is needed to determine if Landsat 8 can accurately capture pinyon and juniper phenology at seasonal timescales while considering effects from vegetation within the interspace.

For the meadow and sagebrush communities, the agreement between phenocam NDVI and Landsat NDVI was greatest at POP, then SOS and with less agreement at EOS during fall senescence. However, in this study there was no consistent time trend between Landsat and phenocam threshold dates; either platform could predict earlier or later dates depending on sites and years. The best agreement was in the wet meadow, which not surprisingly has the densest vegetation and least bare ground. Similar to this study, work in eastern deciduous forests at thirteen phenocam sites determined that phenocam-based indices of greenness (GCC) were in greater agreement with MODIS NDVI during spring green-up than during fall senescence [27]. Furthermore in contrast to the current study, MODIS phenophase dates were consistently two to eight days later than phenocam dates [27]. Zhang et al. [31] used 82 phenocam sites across the continental United States and found good agreement between MODIS EVI, VIIRS NDVI and EVI2 values and phenocam GCC and vegetation contrast index (VCI). Similar to this study, indices agreed better during spring green-up than during fall senescence; absolute differences between VIIRS EVI2 and phenocam VCI were 7–11 days in green-up, but increased to 10–13 during fall senescence [31]. The stronger agreement between satellite and phenocam indices during spring is thought to be due to the rapid and more homogenous spring leaf-out, whereas the greater lack of agreement in fall senescence is thought to be due to the more gradual changes in leaf color that encompass more within-canopy and interspace variability. Additionally, phenocam greenness indices are more sensitive to greens than to reds, yellow or browns [27,31], while NDVI is more sensitive to leaf area [26]. In the current study, the discrepancy in autumn between the steady decline in phenocam NDVI versus a small increase then flattening of the Landsat NDVI in the fall in the sagebrush communities could have resulted from phenocam NDVI being less sensitive to the yellow flowers than Landsat. Which is somewhat counterintuitive as Wingate et al. [48] found phenocams GCC to be adept at picking up flowering events in several grassland and crop ecosystems. However, this could reflect important differences in whether to use phenocam-derived GCC or NDVI or some combination of both to describe phenology. Research by Luo et al. [21] suggest that a combination of phenocam indices may provide the most utility.

We were unable to assign phenophase dates to the pinyon and juniper community using Landsat, due to the irregular patterns, which were not able to be fit by a spline curve. However, in several other instances, in the meadow and sagebrush we were unable to determine phenophase dates (Supplementary Information, Table S2). This was likely due to the much smaller number of Landsat dates available to determine phenophase dates. New research by Baumann et al. [49] in deciduous and mixed forest systems of the eastern United States, used dynamic time warping with MODIS data to improve the temporal resolution of Landsat EVI. Results from the Baumann et al. [39] study seem promising, and such an approach could prove useful for other systems where limited cloud free Landsat images are available.

Phenophase dates across the different ROIs generally showed an increased phenocam LOS in the warm, dry year of 2015, which was a result of earlier SOS and later EOS (Supplementary Information, Table S2). The exception to this was the groundwater dependent wet meadow, which showed increased LOS over the three years, likely in response to decreased depth to groundwater in response to the reduced drought conditions (Supplementary Information, Table S2). The mesic meadow also responded differently having a long LOS in 2015 with shorter LOS in 2016 and 2017. After four years of drought the mesic meadow had a shift in species composition to povertyweed, which is characteristic of drier sites and has a long growing season and wide flowering window. With elimination of drought, by 2017 the mesic meadow was dominated by field and Douglas’ sedge, which are characteristic of wetter environments. The shift in species composition was evident in phenocam images and was documented by ground surveys of vegetation composition and basal cover (T.K. Stringham unpublished data). The phenocams were effective in providing complementary information, and more information than that obtained from Landsat alone. Additionally, phenocam data were useful for highlighting the fact that increased NDVI and longer growing seasons are not always providing the same ecosystem goods and services, if weedy species are replacing more desirable species due to decreased water availability and increased temperature. The “greening” effect has been described for the Great Basin with growing seasons lengthening by three days per decade over the last 30 years according to satellite platform NDVI, however the species responsible for that greening cannot be determined at the scale of the analysis (i.e., AVHRR scale at 8 km resolution) [10].

At two of the sites where sagebrush was present (i.e., dry meadow and sagebrush upland) differences in phenocam phenology between the precipitation years were observed. The magnitude and form (rain versus snow) of winter precipitation has been proposed as an important factor in sagebrush habitat suitability throughout its range in the western United States [50,51]. As shown in this study, year to year variation in precipitation, which was mostly snow and subsequent snowmelt, changed the start of season in the sagebrush community (Figure 6, Supplementary Information Table S2). In 2015 sagebrush green-up began 16 and 42 days earlier (Supplementary Information, Table S2), in the upland and dry meadow ROIs, respectively and peak greenness was 15 and 16 days earlier (Figure 6, Figure 8 and Figure 9), in comparison to the wet cold year of 2017. These shifts in phenology coupled with projected changes in climate could create phenological disconnects in a variety of ecosystems. For example, the greater sage grouse (*Centrocercus urophasianus)* is considered a near threatened species. For plant and consumer interactions to be successful, phenology patterns must overlap [11]. Young sage grouse chicks depend on consuming insects and forbs associated with sagebrush and meadow ecosystems. [52]. Early brood rearing habitat (March to June) requires forbs, as a direct source of food and as an indirect source of food by attracting insects; forbs and insects are the main components of sage grouse chicks’ diets during their first few weeks [53]. Monitoring phenology of foundational plant species across variable weather years and with changing climate can provide early warnings of potential phenological disconnects.

In summary, for short statured sagebrush and meadow communities phenocam and Landsat NDVI were in good agreement. In the tall-statured pinyon and juniper woodlands phenocam and Landsat NDVI were in poor agreement. Phenocams can provide a link between satellite observations of plant vigor and greenness and land-based observations of plant vigor and greenness. The increased temporal resolution of phenocam provides important information for informing management decisions such as how to properly utilize forage, manage for wildlife habitat, and control invasive weeds [47]. Combining phenocam and Landsat vegetation metrics has the potential for greater spatio-temporal coverage at field and landscape-scales, and greatly extends the historic record allowing management decisions to be viewed in a broader context.

## Figures and Tables

**Table 1:** Camera orientation data which includes the height from ground, angle view from horizontal to the ground surface, azimuth and aspect. The regions of interest (ROI)s from Figure 2 and Figure 3 are as follows: Camera (1) three ROIS-pinyon and juniper woodland, large pinyon and juniper woodland and valley sagebrush in Figure 2; Camera (2) One ROI the upland sagebrush in Figure 3a; Camera (3) three ROIS-wet meadow, mesic meadow and dry meadow in Figure 3b.

Camera Site	Height (m)	Angle (°)	Azimuth (°)	Aspect
1. Pinyon and juniper Woodland & Valley Sagebrush	1.85	0.6	294	WNW
2. Upland Sagebrush	2.18	8.2	250	WSW
3. Meadow	2.28	−0.9	205	SSW

**Table 2:** Root Mean Square Error (RMSE) between phenocam and Landsat scaled NDVI.

Year	Pinyon & Juniper Woodland	Pinyon & Juniper Interspace	Upland Sagebrush	Valley Sagebrush	Dry Meadow	Mesic Meadow	Wet Meadow
2015	0.44	0.39	0.18	0.07	0.12	0.15	0.11
2016	0.43	0.24	0.13	0.10	0.07	0.14	0.10
2017	0.41	0.18	0.17	0.10	0.15	0.13	0.11
All Years	0.42	0.26	0.16	0.09	0.12	0.14	0.11

**Table 3:** Pearson’s Correlation Coefficient (*r*) of phenocam and Landsat scaled NDVI.

Year	Pinyon & Juniper Woodland	Pinyon & Juniper Interspace	Upland Sagebrush	Valley Sagebrush	Dry Meadow	Mesic Meadow	Wet Meadow
2015	−0.53	0.22	0.84	0.84	0.78	0.76	0.92
2016	−0.40	0.34	0.88	0.93	0.98	0.87	0.93
2017	−0.25	0.44	0.87	0.90	0.90	0.93	0.98
All Years	−0.24	0.22	0.86	0.92	0.93	0.84	0.96

**Table 4:** Landsat and phenocam actual minimum and maximum NDVI values and the seasonal amplitude between the minimum and maximum values. These are the actual calculated values, not the rescaled values presented in the figures. Bolded values are the amplitude values from the two platforms.

Site & Years	Landsat Min	Landsat Max	Landsat Amplitude	Phenocam Min	Phenocam Max	Phenocam Amplitude
*Pinyon & Juniper Woodland* *2015* *2016* *2017*						
	0.30	0.41	**0.11**	−0.14	−0.08	**0.06**
	0.32	0.42	**0.10**	−0.16	−0.06	**0.10**
	0.32	0.39	**0.07**	−0.12	−0.06	**0.06**
*Upland Sagebrush* *2015* *2016* *2017*						
	0.22	0.35	**0.13**	−0.34	−0.24	**0.10**
	0.19	0.48	**0.29**	−0.33	−0.12	**0.21**
	0.13	0.42	**0.28**	−0.34	−0.21	**0.13**
*Valley Sagebrush* *2015* *2016* *2017*						
	0.21	0.30	**0.09**	−0.37	−0.24	**0.13**
	0.18	0.40	**0.21**	−0.41	−0.12	**0.30**
	0.26	0.36	**0.11**	−0.38	−0.19	**0.19**
*Dry Meadow* *2015* *2016* *2017*						
	0.21	0.31	**0.10**	−0.36	−0.19	**0.17**
	0.15	0.41	**0.26**	−0.38	−0.07	**0.31**
	0.17	0.38	**0.21**	−0.35	−0.08	**0.27**
*Mesic Meadow* *2015* *2016* *2017*						
	0.23	0.39	**0.16**	−0.30	0.00	**0.30**
	0.16	0.49	**0.33**	−0.32	0.17	**0.49**
	0.18	0.55	**0.37**	−0.39	0.15	**0.55**
*Wet Meadow* *2015* *2016* *2017*						
	0.19	0.52	**0.34**	−0.24	0.09	**0.33**
	0.19	0.62	**0.43**	−0.36	0.22	**0.58**
	0.18	0.76	**0.58**	−0.33	0.32	**0.65**

## Supplementary Materials

The following are available online at https://www.mdpi.com/1424-8220/19/5/1139/s1, Figure S1: (a) Training and testing points for nnet classification, and (b)The nnet 2 class model classification. Table S1: A list of commonly used abbreviations. Table S2: Length of Season (LOS) for site year which is the difference between start of season (SOS) and end of season (EOS) from Landsat and phenocam derived phenophase dates.
