# Supplementary material for: Comparison of Landsat and Land-Based Phenology Camera Normalized Difference Vegetation Index (NDVI) for Dominant Plant Communities in the Great Basin
Source: Sensors (Basel). 2019 Mar 6;19(5):1139. doi: 10.3390/s19051139 (PMC6427513; doi:10.3390/s19051139)
Supplement: Supplementary file 1 [file sensors-19-01139-s001.pdf]

sensors

MDPI

Supplementary Information

Figures and Table not shown in the main manuscript.

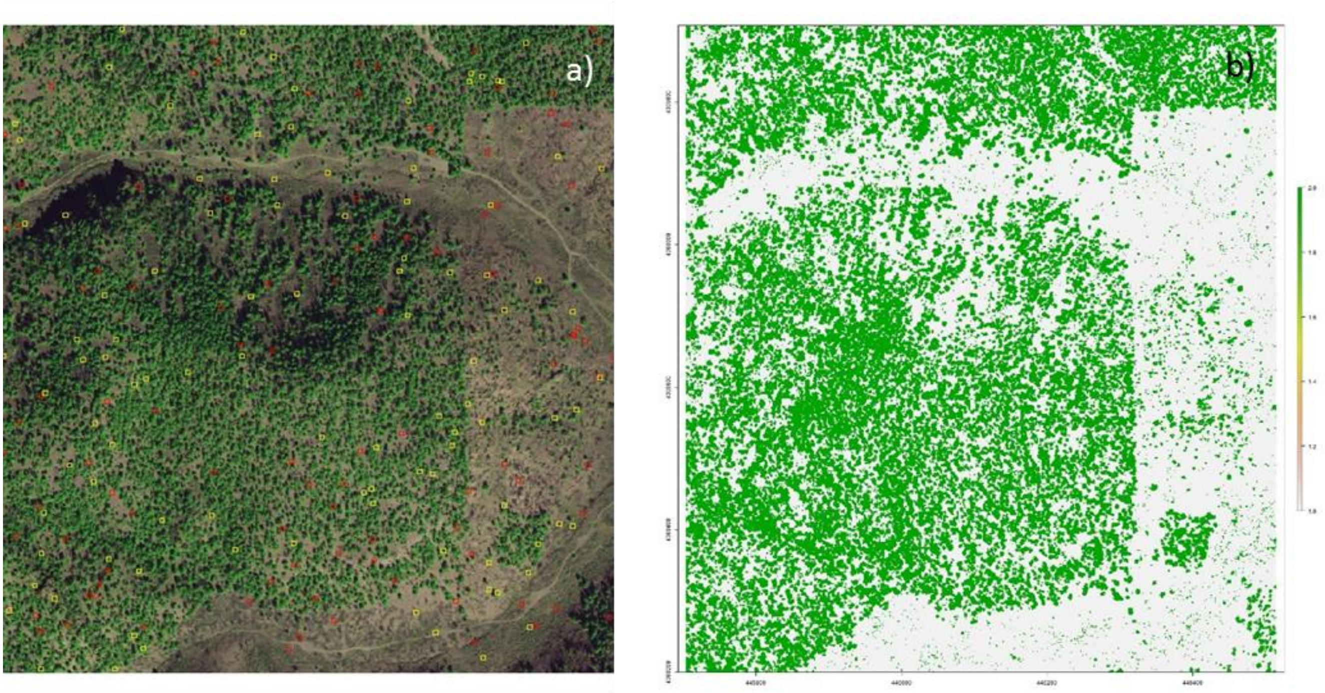

Figure S1. **(a)** Training and testing points for *nnet* classification, and **(b)** The *nnet* 2 class model classification.

sensors

MDPI

Table S1. Length of Season (LOS) for site year which is the difference between start of season (SOS) and end of season (EOS) from Landsat and phenocam derived phenophase dates.

Site Years	Landsat SOS	Phenocam SOS	Landsat EOS	Phenocam SOS	Landsat LOS	Phenocam LOS
Pinyon & Juniper Woodland						
2015	NA	106 (45-206)	NA	292 (94-302)	NA	182 (91-251)
2016	NA	176 (140-176)	NA	254 (249-298)	NA	78 (73-158)
2017	NA	70 (68-78)	176 (156-247)	200 (144-252)	NA	130.5 (76-183)
Upland Sagebrush						
2015	130 (130-130)	96 (77-99)	210 (210-210)	207 (205-246)	80 (80-80)	110 (107-169)
2016	110 (94-110)	108 (107-110)	197 (197-222)	230 (228-276)	87 (87-103)	122 (118-168)
2017	87 (87-87)	112 (108-114)	192 (188-220)	224 (223-272)	105 (101-109)	112 (109-164)
Valley Sagebrush						
2015	91 (75-107)	120 (96-124)	235 (235-251)	240 (238-262)	160 (128-176)	120 (115-166)
2016	110 (94-133)	130 (114-134)	202 (181-210)	236 (232-275)	92 (64-112)	104 (98-161)
2017	NA (NA-NA)	124 (118-127)	207 (199-215)	223 (220-278)	NA	99 (93-158)
Dry Meadow						
2015	130 (130-130)	86 (78-94)	210 (210-210)	272 (270-272)	80 (80-80)	184 (178-193)
2016	102 (94-122)	108 (96-109)	234 (210-262)	238 (232-284)	140 (96-167)	131 (124-186)
2017	119 (119-119)	128 (102-129)	236 (220-244)	236 (235-286)	117 (109-125)	108 (107-184)
Mesic Meadow						
2015	130 (130-130)	94 (84-100)	NA	246 (245-259)	NA	152 (146-175)
2016	110 (110-110)	102 (99-103)	206 (197-218)	198 (194-252)	96 (87-96)	97 (92-155)
2017	128 (119-128)	113 (108-117)	240 (228-247)	218 (213-276)	116 (103-125)	105 (96-165)
Wet Meadow						
2015	130 (130-130)	132 (94-134)	NA	239 (235-262)	NA	107 (101-168)
2016	133 (133-133)	122 (114-126)	286 (229-298)	257 (251-289)	157 (112-165)	136 (126-163)
2017	128 (119-128)	124 (114-128)	236 (220-244)	276 (267-298)	108 (96-119)	152 (139-181)

sensors

MDPI

Table S2. A list of commonly used abbreviations.

**Abbreviations**	**Description**
BCC	Blue Chromatic Coordinate derived from phenocam RGB images
DN	Digital Number from images
GCC	Green Chromatic Coordinate derived from phenocam RGB images
MODIS	Moderate Resolution Imaging Spectroradiometer, a satellite platform
NAIP	National Agricultural Imagery Program from aerial imagery
NDVI	Normalized Difference Vegetation Index derived from phenocam and satellite platforms
NIR	Near-infrared reflectance derived from phenocams and satellite platforms
RGB	Standard Red, Green, and Blue color images
ROI	A Region of Interest defined on phenocam images
SOS, POP & EOS	Threshold dates which are Start of Season, Peak of Production, and End of Season
LOS	Length of Season which is the difference between SOS and EOS
